# Effect of partial hysterectomy on the neurons of the paracervical ganglion (PCG) of the pig

**DOI:** 10.1371/journal.pone.0245974

**Published:** 2021-01-26

**Authors:** Piotr Podlasz, Krzysztof Wasowicz

**Affiliations:** Department of Pathophysiology, Forensic Veterinary Medicine and Administration, Faculty of Veterinary Medicine, University of Warmia and Mazury in Olsztyn, Olsztyn, Poland; National Center for Toxicological Research, UNITED STATES

## Abstract

Autonomic neurons innervating uterine horn is probably the only nerve cell population capable of periodical physiological degeneration and regeneration. One of the main sources of innervation of the uterus is paracervical ganglion (PCG). PCG is a unique structure of the autonomic nervous system. It contains components of both the sympathetic and parasympathetic nervous system. The present study examines the response of neurons of PCG innervating uterine horn to axotomy caused by partial hysterectomy in the domestic pig animal model. The study was performed using a neuronal retrograde tracing and double immunofluorescent staining for tyrosine hydroxylase (TH), dopamine beta-hydroxylase (DβH), choline acetyltransferase (ChAT), vesicular acetylcholine transporter (VAChT), neuronal nictric oxide synthase (nNOS), galanin, neuropeptide Y (NPY), vasoactive intestinal peptide (VIP), pituitary adenylate cyclase-activating peptide (PACAP), somatostatin and substance P (SP). Our study showed that virtually all neurons of the porcine PCG innervating uterine horn are adrenergic and we did not confirm that PCG is the source of cholinergic fibers innervating uterine horn of the pig. After axotomy there was a decrease in expression of catecholamine-synthesizing enzymes (TH, DβH) and a strong increase in the galanin expression. The increase of the number of NPY-IR neurons in the ganglia after axotomy was observed. There were no changes in the expression of other studied substances in the PCG neurons innervating the uterine horn, what was often found in rodents studies. This indicates that neurons can respond to damage in a species-specific way.

## Introduction

The female pelvic plexus contains numerous nerve ganglia located in the parametrium at the utero-vaginal junction. This collection of ganglia is termed the paracervical ganglion (PCG). Postganglionic nerve fibers originating from PCG are responsible for the innervation of the lower urinary and digestive tract as well as reproductive organs including uterus [1, 2]. PCG is a unique structure of the autonomic nervous system. It contains components of both the sympathetic and parasympathetic pathways [1, 3]. Adrenergic neurons of PCG are called "short" adrenergic neurons, in contrast to "long" adrenergic neurons, located in pre- and paravertebral ganglia [4]. Physiological studies suggest that non-adrenergic neurons located in the ganglia of the female pelvic plexus are responsible for a variety of cholinergic actions, including vasodilatation of the uterine arteries [5]. In the reproductive system, the plasticity of the nervous system is associated with the changes in uterine innervation density occurring during the course of the estrous cycle and pregnancy [6–11].

Particularly significant changes in the uterine innervation were observed during the pregnancy. The noradrenaline (NA) content decreased significantly in the uterine horn of the guinea pig, reaching the lowest levels just before delivery [12]. At the same time, the adrenergic nerve fibers were progressively lost so that at the end of the pregnancy they were virtually undetectable by histochemical methods [8, 9]. After delivery, the density of the adrenergic innervation in the uterine horn was slowly returning to its initial state [13]. Changes in the cholinergic innervation of the uterine horn were of different nature. The number of cholinergic nerve fibers in the uterine horn decreased during pregnancy, but after delivery, in contrast to adrenergic fibers, it was returning quickly to normal [6]. In these fibers, there were no clear signs of degenerative processes described in the adrenergic fibers.

Regeneration of the adrenergic nerve fibers in the uterine horn after delivery indicates that the adrenergic neurons innervating the uterine horn are probably the only nerve cell population capable of periodical physiological degeneration and regeneration.

One of the causes of neuronal degeneration is neurotrophic factors deprivation. It can be achieved by axotomy [14], nerve crush, axonal transport blocking agents, e.g. colchicine [15] or acrylamide [16], or the use of the neurotrophin-scavenging antibodies [17].

The main goal of the study was to create an experimental system that would allow to study the plasticity of neurons innervating the uterus. Most of the studies of plasticity of the autonomic neurons was done on the rodent models. However, rodents often respond to experimental interventions in ways that differ strikingly from that of humans [18]. To compare the data obtained in a rodent model we decided to use the domestic pig, the species more comparable in size, anatomy, physiology, metabolic rate and diets to human than rodents. The distribution and immunohistochemical characteristics of neurons responsible for the innervation of the reproductive organs in the pig was studied in great detail [2, 19–26] and this species becomes increasingly important as a model animal in biomedical studies [27]. PCG was chosen for the study as a potential source of both adrenergic and cholinergic innervation of uterus. As described above the neurons in the PCG innervating uterus has unique properties and the mechanisms of periodic degeneration and regeneration in the course of the estrous cycle and pregnancy is still unclear. Besides, pelvic neurons are unusually susceptible to axonal injury. Tumors, trauma and iatrogenesis during various surgical procedures like hysterectomy have all been identified as potential causes of pelvic plexus injury [28]. It was decided to investigate the reaction of the neurons to axotomy caused by uterine horn extirpation in sexually immature animals to avoid potential interference from the endocrine factors associated with an estrus cycle. The study may be an introduction to further studies on the reaction of PCG neurons on inflammation of the uterus and other pelvic organs, caused by various pathological factors such as microbiological, chemical (e.g. toxins) and physical (e.g. ionizing radiation) factors.

## Materials and methods

The experiment was approved by the Local Ethics Committee in Olsztyn, Poland (no. 37/2002/N) affiliated to the National Ethics Commission for animal experimentation (Polish Ministry of Science and Higher Education).

**Fig 1:**
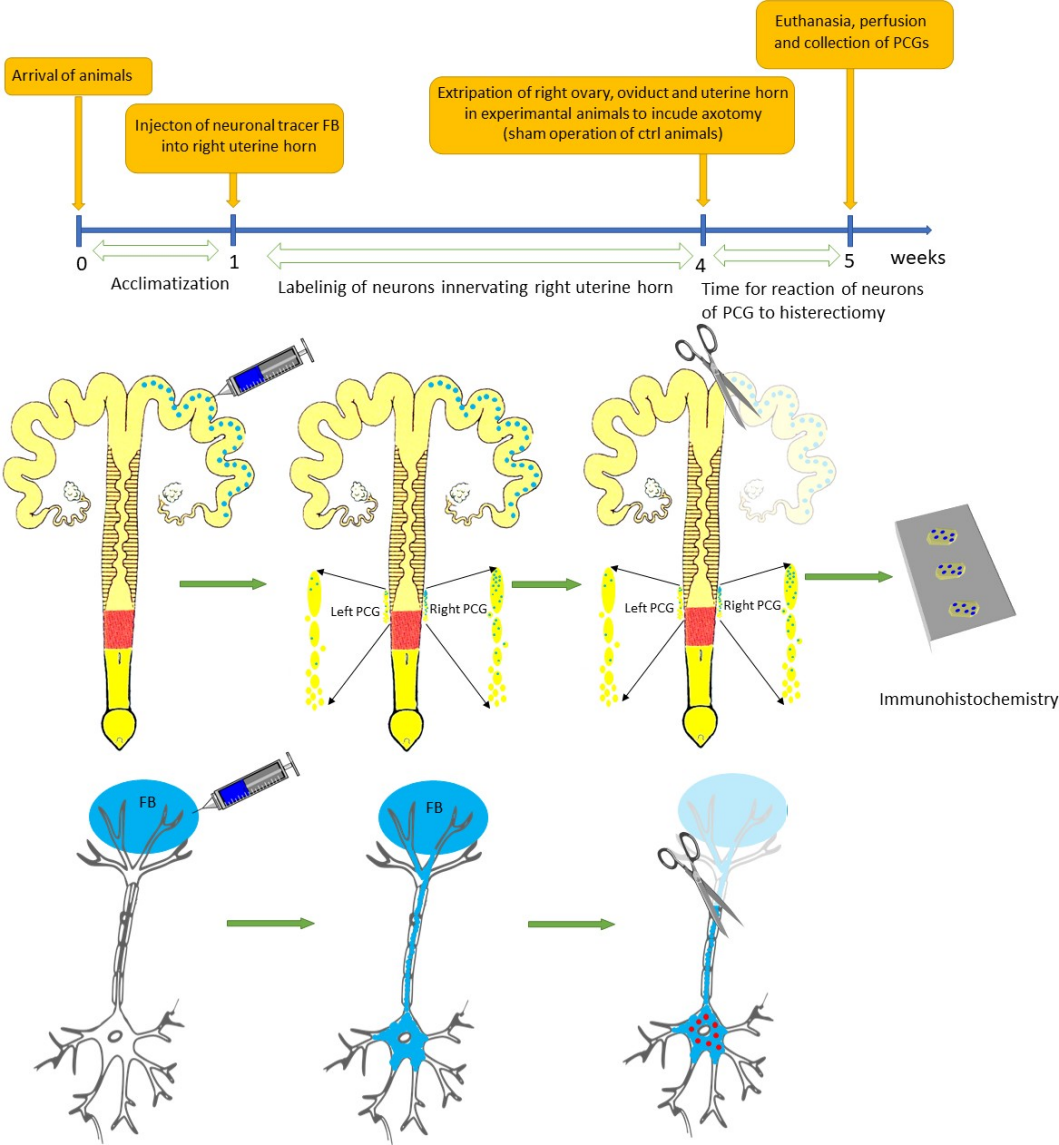
Timeline and flowchart of neuronal tracing of neurons of PCG innervated of uterine horn and partial hysterectomy used in the study.

Ten sexually immature gilts (body weight of ca. 20 kg) were used for the study. The animals originated from the commercial fattening farm. They were kept under standard conditions with a free access to water. Animals were operated as described before [29]. In all animals 5% suspension of fluorescent tracer Fast Blue (FB, (Polysciences, Inc) in the total volume of 50 μl of water was injected into multiple sites spaced evenly on the length of right uterine horn on the side opposite to the ligamentum latum uteri (Fig 1). To minimize leakage of FB the needle of the syringe was left in place for 1 min. The wall of the uterus was subsequently rinsed with physiological saline and gently wiped with gauze. After 3 weeks 5 animals underwent second laparotomy under general anesthesia as described before [25], during which the right uterine horn with ovary and oviduct was extirpated. In remaining 5 animals only sham operation was performed. After one week all animals were sacrificed with a lethal dose of pentobarbital and perfused transcardially with ca. 1.5 liter of 4% paraformaldehyde in 0.1M phosphate buffer (pH 7.4, PFA). Uterine cervices and vaginas were removed and postfixed in the same fixative for 30 minutes. They were then placed in 18% sucrose in 0.1M phosphate buffer and stored at +4°C until they sank to the bottom of the container. 20 μm cryostat sections were put on chrome alum-gelatin-coated slides, allowed to dry and stored desiccated at –70°C until processing.

The slides were rehydrated in phosphate-buffered saline (PBS, pH 7.4) and processed for double-immunofluorescence. The slides were washed and preincubated in PBS containing Triton X-100 0.1% (PBS-T, pH 7.4), 0.1% sodium azide, and 4% normal goat serum (NGS) for 1 h at RT. Next, the slides were incubated with the primary antibodies (Table 1) in the preincubation solution for 14–24 h at RT. Afterward, they were washed thoroughly with PBS-T and incubated with an appropriate biotinylated antiserum (1 h, RT) followed by appropriate fluorescein isothiocyanate (FITC)-conjugated secondary antiserum or CY3-conjugated streptavidin (Table 1) in the preincubation solution for 1 h at RT. After extensive washing with PBS, the specimens were mounted in 80% glycerol in PBS.

**Table 1:** Antibodies used in the study.

Antigen	Species	Code	Dilution	Supplier
PRIMARY ANTIBODIES				
tyrosine hydroxylase (TH)	mouse	2/40/15	1:120	Boehringer, Mannheim, Germany
dopamine beta-hydroxylase (DβH)	rabbit	DZ1020	1:2000	Affiniti, Mamhead, UK
choline acetyltransferase (ChAT)	rabbit	AB143	1:5000	Chemicon, Temacula, USA
(vesicular acetylcholine transporter) VAChT	rabbit	G4481	1:5000	Promega, Medison, USA
neuronal nictric oxide synthase (nNOS)	rabbit	210–504	1:5000	Alexis, Lausen, Switzerland
Neuropeptide Y (NPY)	rabbit	RNP1702	1:5000	Amersham, Buckinghamshire, UK
vasoactive intestinal peptide (VIP)	mouse	MaVIP	1:1500	East Acres, Southbridge, USA
pituitary adenylate cyclase-activating peptide (PACAP)	rabbit	IHC 8922	1:20000	Peninsula, San Carlos, USA
Galanin	rabbit	Rin-7153	1:1600	Peninsula, San Carlos, USA
Somatostatin	rat	YC7	1:100	Serva, Heidelberg, Germany
Substance P (SP)	rat	RPN 1572	1:700	Amersham, Buckinghamshire, UK
SECONDARY REAGENTS				
FITC-coniug. goat anti-mouse IgG		55493	1:400	Cappel, Durham, USA
FITC-coniug. goat anti-rat IgG		55745	1:400	Cappel, Durham, USA
Biotinyl. goat anti-rabbit IgG		E 0432	1:400	Dako, Glostrup, DK
Streptavidin-CY3		016-160-084	1:4000	Jackson Immunoresearch Lab. Inc., USA

Careful controls for antiserum specificity were carried out. Preabsorption controls of antisera were done by preincubating the antibody with the specific immunogen (10 μg/ml) for 24 h under slow stirring at 4°C. Negative controls were performed by omitting all primary or secondary antibodies in the staining protocol. In all controls immunostaining was completely eliminated.

For image documentation of results, a confocal microscope (Bio-Rad Microradiance MRA 2) and a digital camera (Olympus Camedia C-2020) coupled with a fluorescence microscope (Axiophot, Zeiss, Germany) were used. For this purpose, the FB+ neurons were photographed using a digital camera on the double-stained section and then this region was scanned using a confocal microscope using the LaserSharp 2000 v. 3.1 program (Bio-Rad) using a filter set for FITC (HQ 500 LP) and Texas Red / Cy3 (E 600 LP). For every sample 10 optical section were taken with 1μm Z spacing. The stack of the images obtained with the confocal microscope were compiled to produce maximum intensity projection images and two channels were combined using the LaserPix program v. 4.0013 (Media Cybernetics L.P.) to analyze the colocalization of nerve structures.

FB-positive neurons containing immunoreactivity for the studied neurotransmitters and neurotransmitters synthesizing enzymes were counted with fluorescence microscope (Axiophot, Zeiss, Germany). For each staining, at least 250 FB-positive neurons from each animal were counted. Only neurons with the visible nucleus were counted. The data were presented as percentages (mean ±SEM, N = 5) of FB-positive neurons containing immunoreactivities to the particular studied substances. Results were analyzed by Wilcoxon signed-rank test (Statistica 13.3, TIBCO Software Inc.).

## Results

### Distributions of FB+ neurons

FB-containing neurons were found in both right and left PCG. Their number in the left PCG was much lower. FB + neurons usually appeared in groups of several, a dozen or so, but often were also located alone among the FB-negative neurons. The majority of FB + cells occurred in the ganglia located under the serous membrane of the uterine cervix and in the adventitia of the cranial part of the vagina. Number of the FB+ neurons gradually decreased in the caudal direction.

### Immunohistochemical characterization of FB + neurons

**Fig 2:**
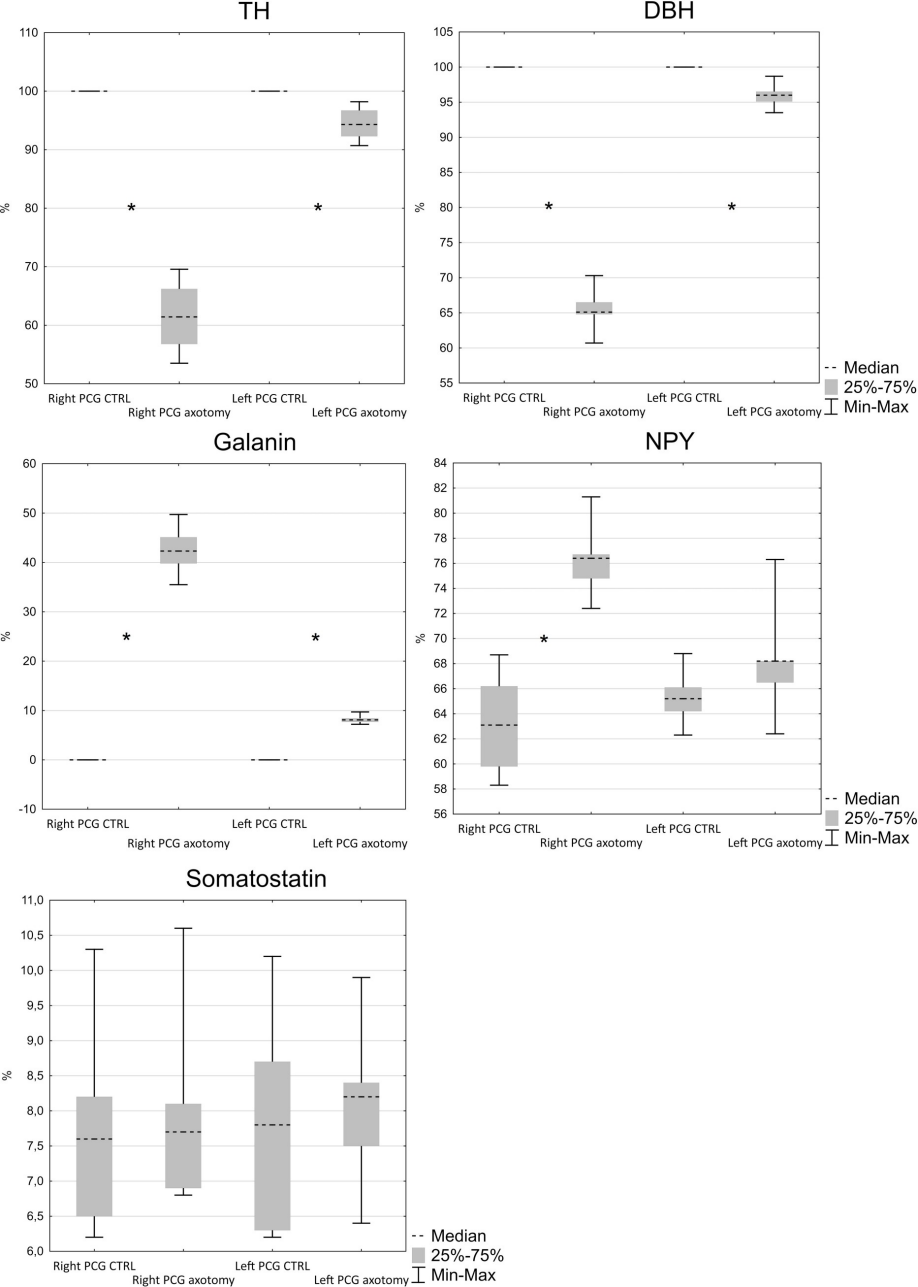
Number of TH, DβH, galanin, NPY and somatostatin-immunoreactive neurons in FB+ neurons in PCG. (N = 5, Z = 2.02, p < 0.05).

**Fig 3:**
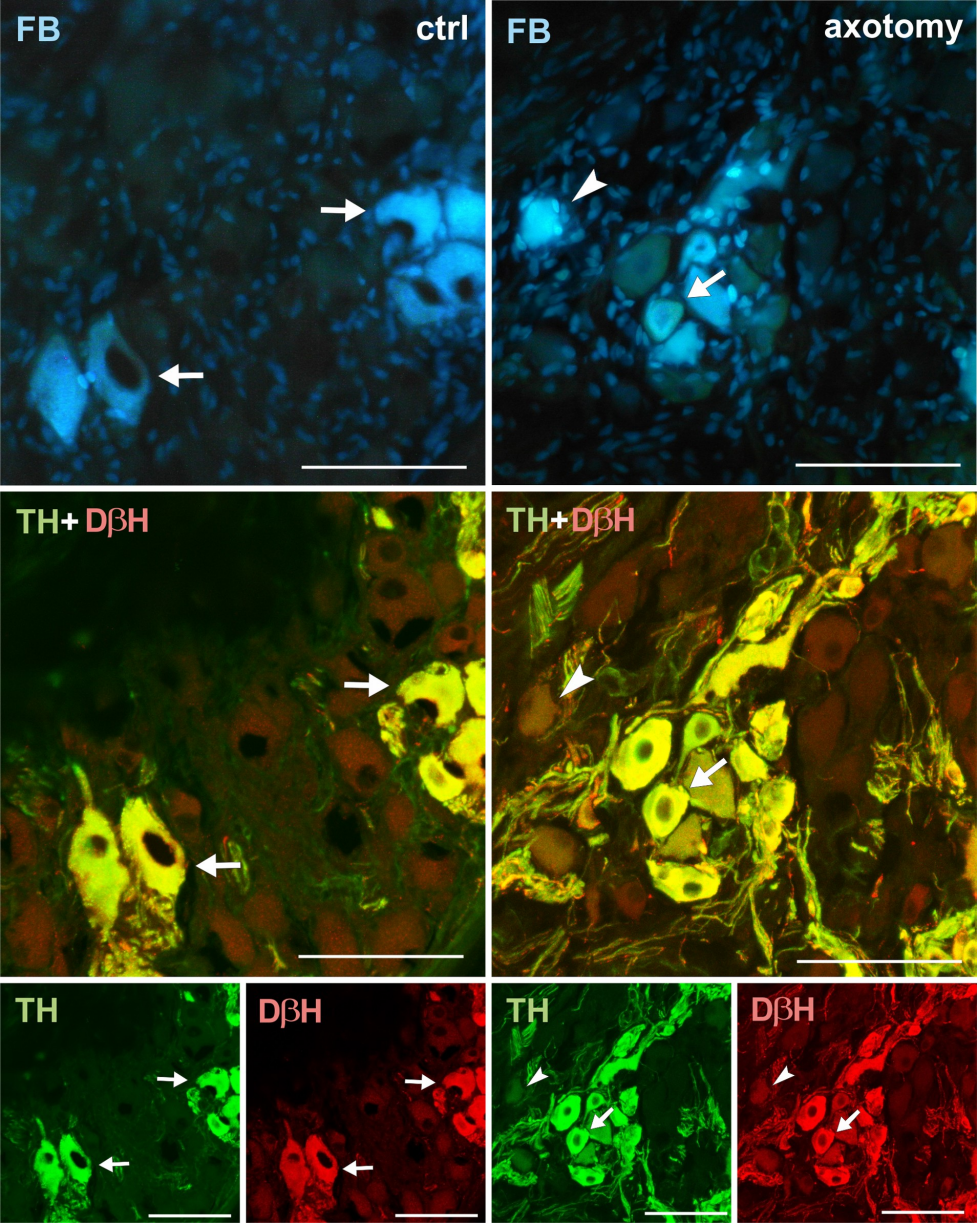
Changes in the expression of TH and DβH in PCG neurons after hysterectomy. All FB + neurons showed TH and DβH immunoreactivity in control animals (arrows). After hysterectomy in some of the FB+ neurons weak or lack of immunoreactivity for TH and DβH were observed (arrowhead). Scale bars 50 μm.

**Fig 4:**
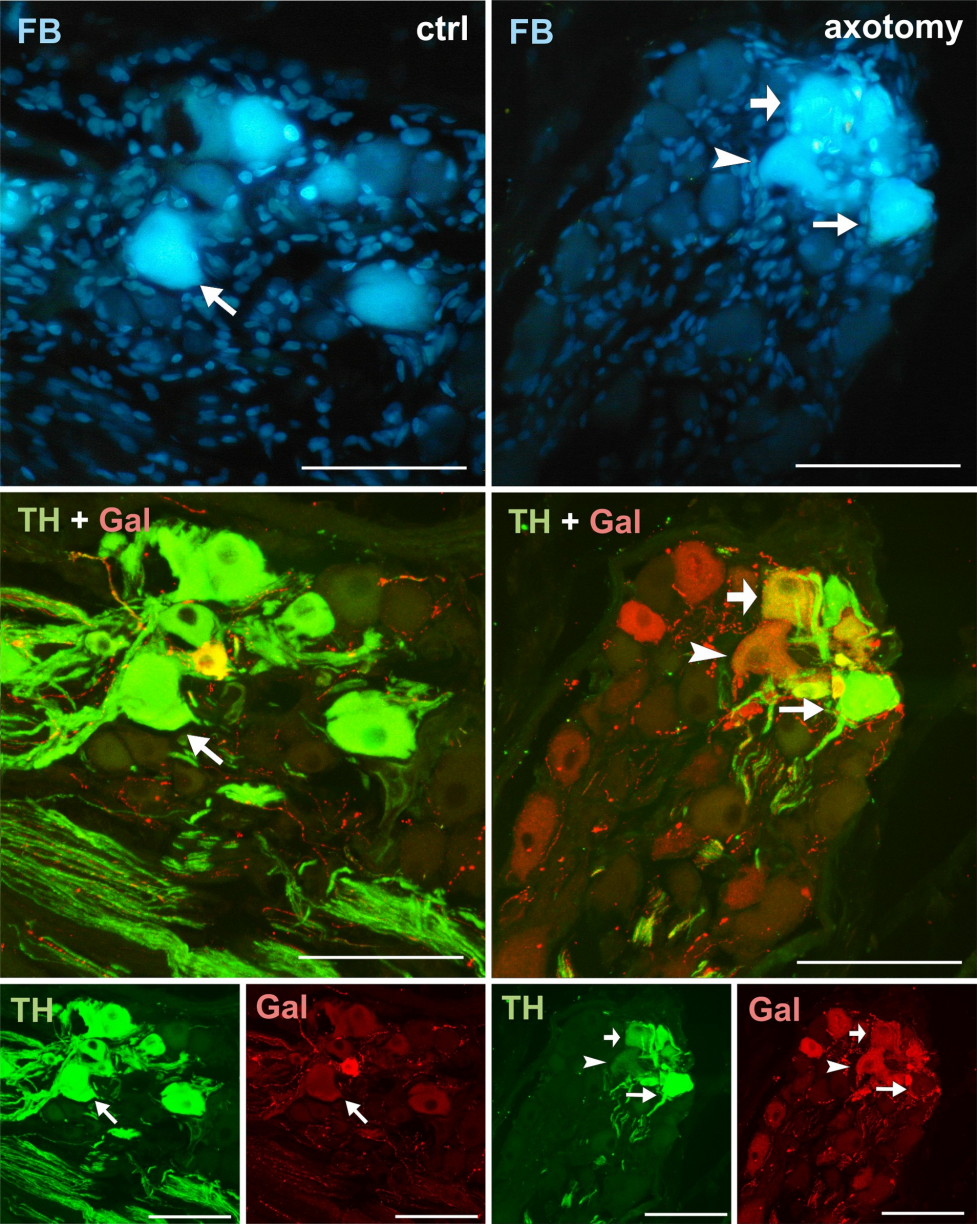
Changes in the expression of TH and galanin in PCG neurons after hysterectomy. All FB+ neurons showed TH immunoreactivity, but none contained galanin-IR in control animals (arrows). After hysterectomy among FB+ neurons, TH- / galanin + (arrowhead) and TH+/galanin+ (thick arrow) were observed. Scale bars 50 μm.

**Fig 5:**
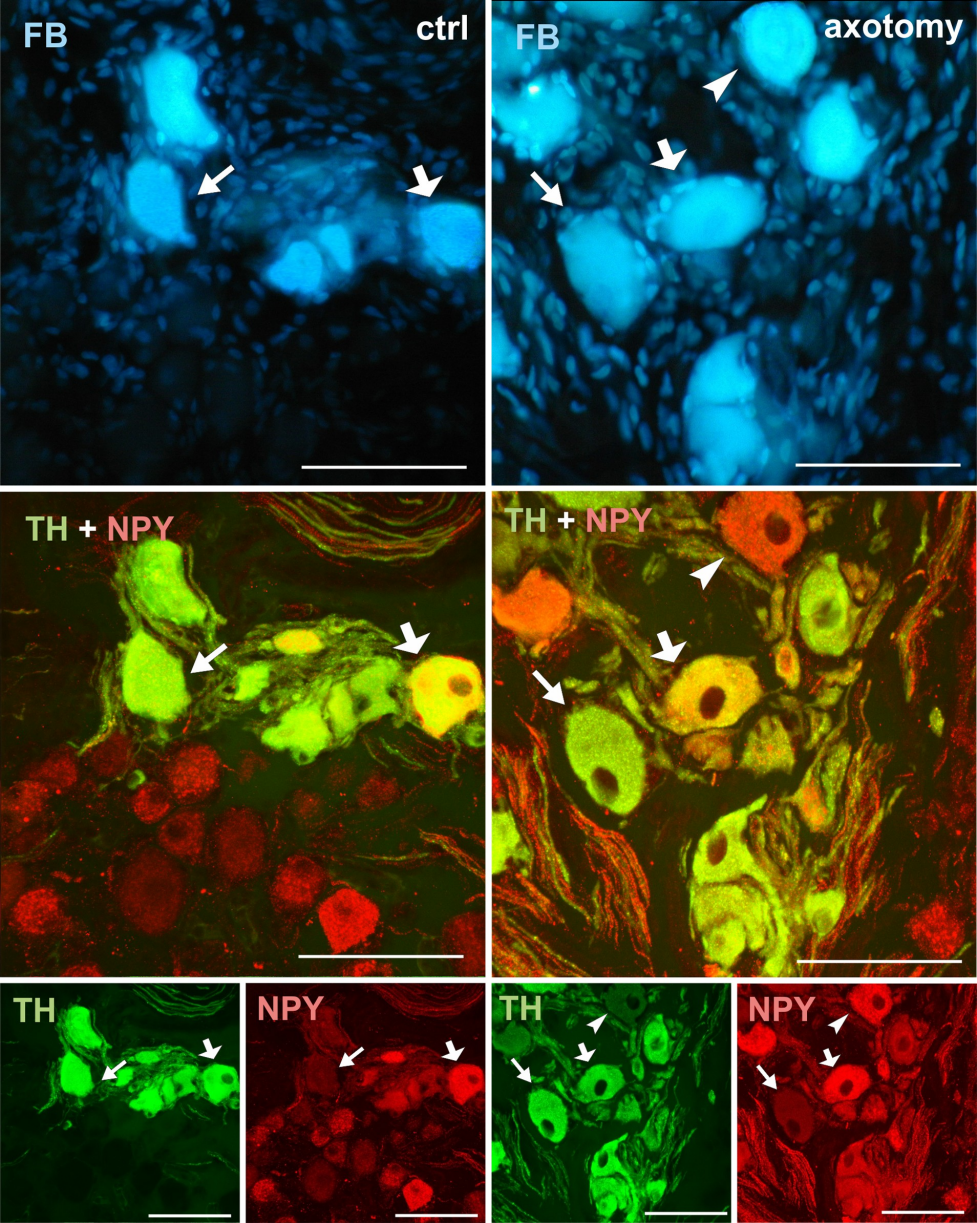
Changes in the expression of NPY in PCG neurons after hysterectomy. Double-stained PCG sections with antibody against TH and NPY. In the control group all of the FB+ neurons contain immunoreactivity to TH. Most of them contained NPY-IR (thick arrow), but some of them were NPY negative (arrow). After hysterectomy the number of NPY-IR neurons slightly increased. Also some of the TH-/NPY+ (arrowhead) and TH-/NPY- neurons were observed. Scale bars 50 μm.

**Fig 6:**
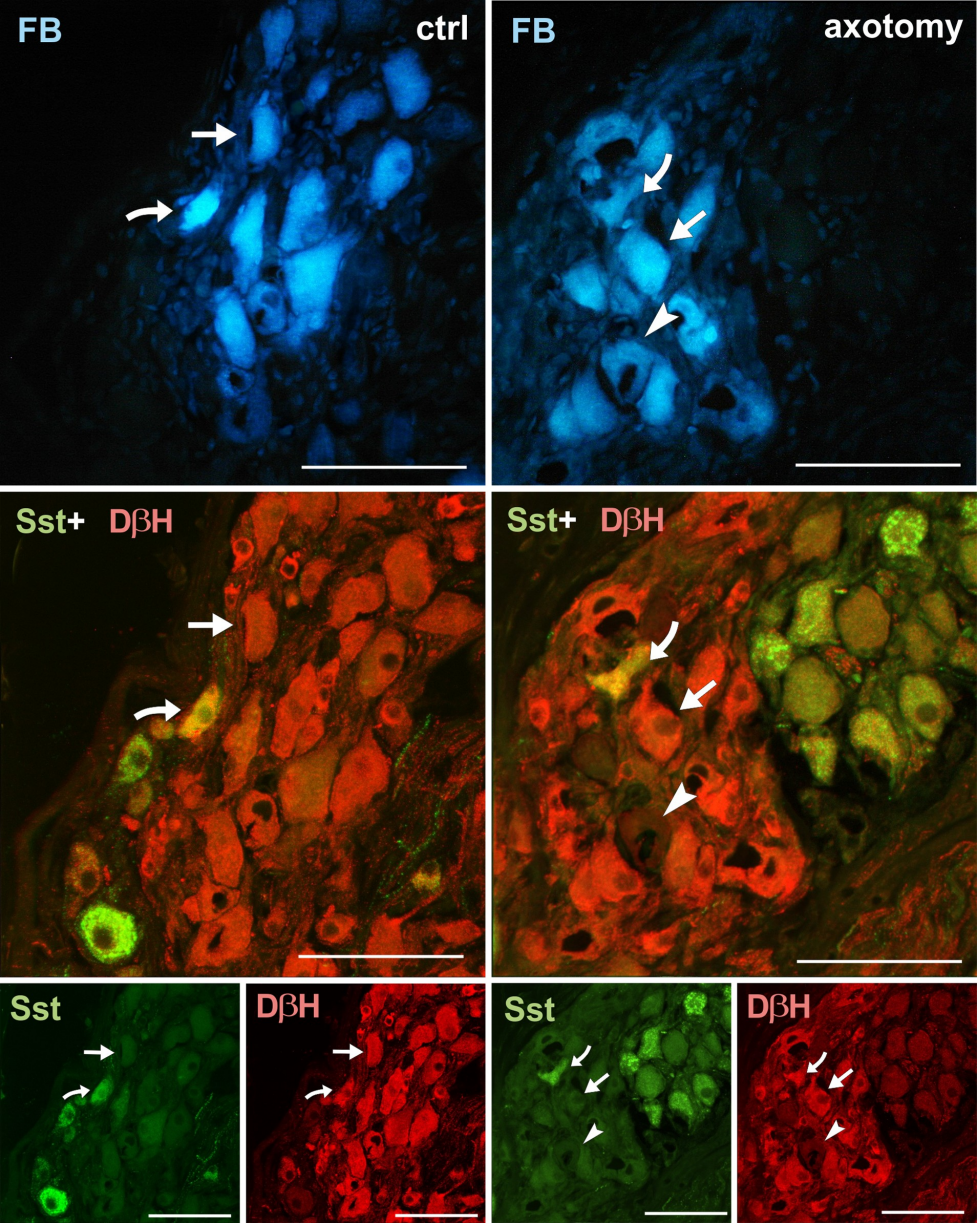
Changes in the expression of somatostatin (Sst) in PCG neurons after hysterectomy. Sections of PCG sections immunolabelled with DβH and somatostatin antibody. In the control group, FB+ neurons were usually DβH+ and somatostatin negative (arrows), less often DβH+/Sst+ (curved arrow). After hysterectomy some DβH-/Sst- (arrowhead) neurons were observed. DβH-/Sst+ neurons appeared very rarely. Scale bars 50 μm.

**Fig 7:**
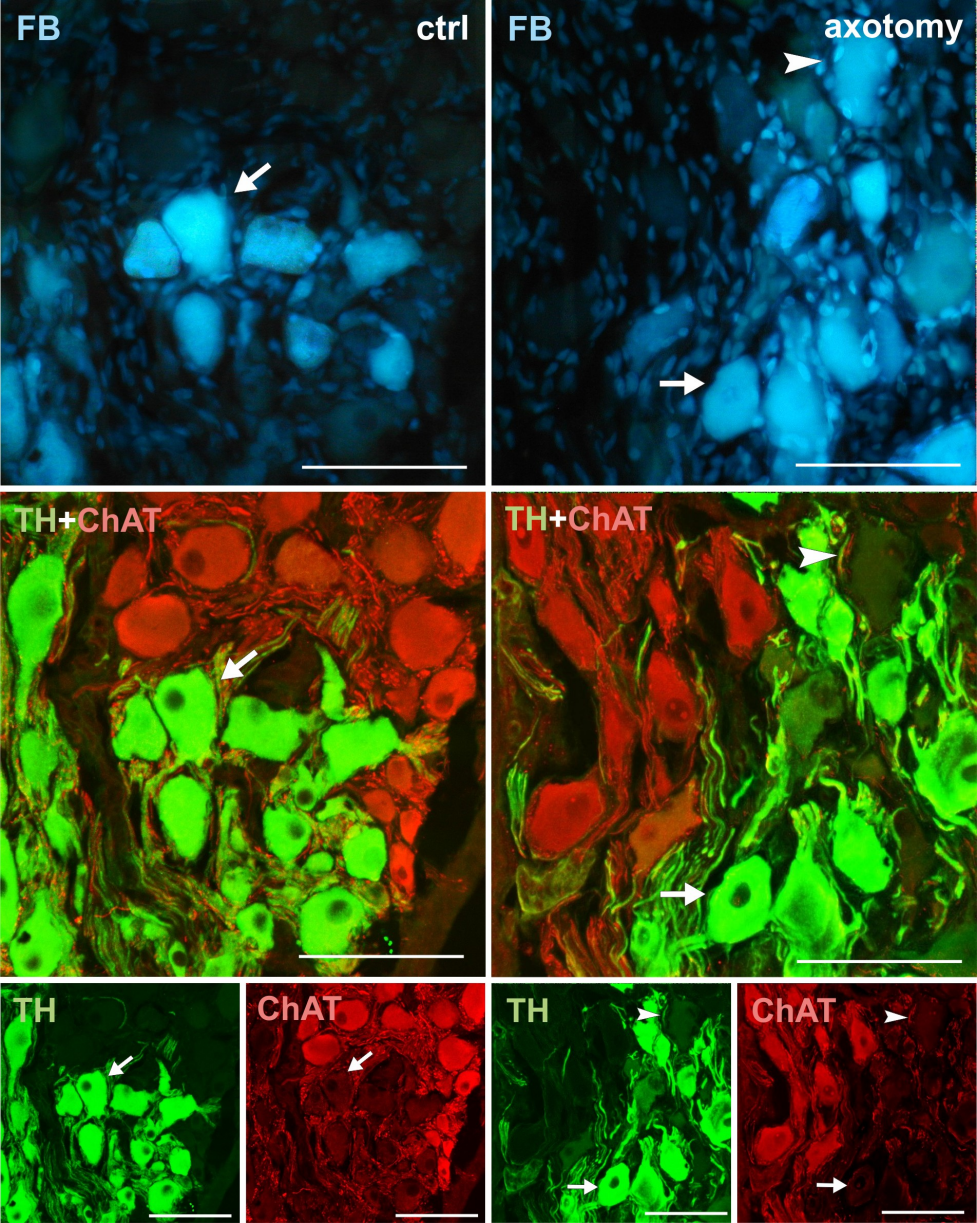
Changes in the expression of ChAT in PCG neurons after hysterectomy. In the control group all FB + neurons contained TH and did not contain ChAT immunoreactivity (arrows). After hysterectomy, some neurons not containing TH nor ChAT immunoreactivity were observed (arrowhead). Scale bars 50 μm.

**Fig 8:**
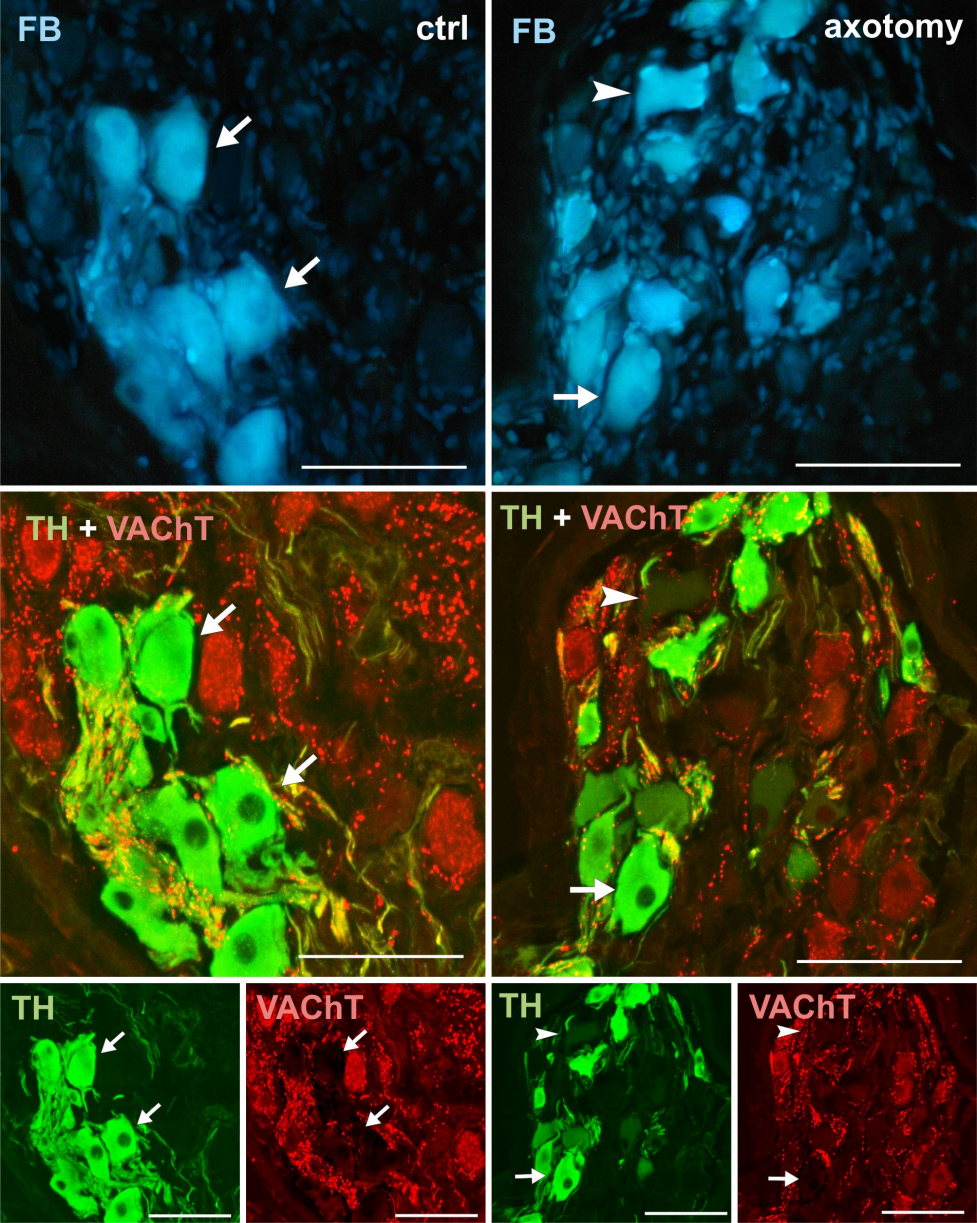
Changes in the expression of VAChT in PCG neurons after hysterectomy. In the control group all FB+ neurons contained TH and did not contain VAChT (arrows). After hysterectomy population of neurons not containing TH nor VAChT (arrowhead) also were visible. Scale bars 50 μm.

**Fig 9:**
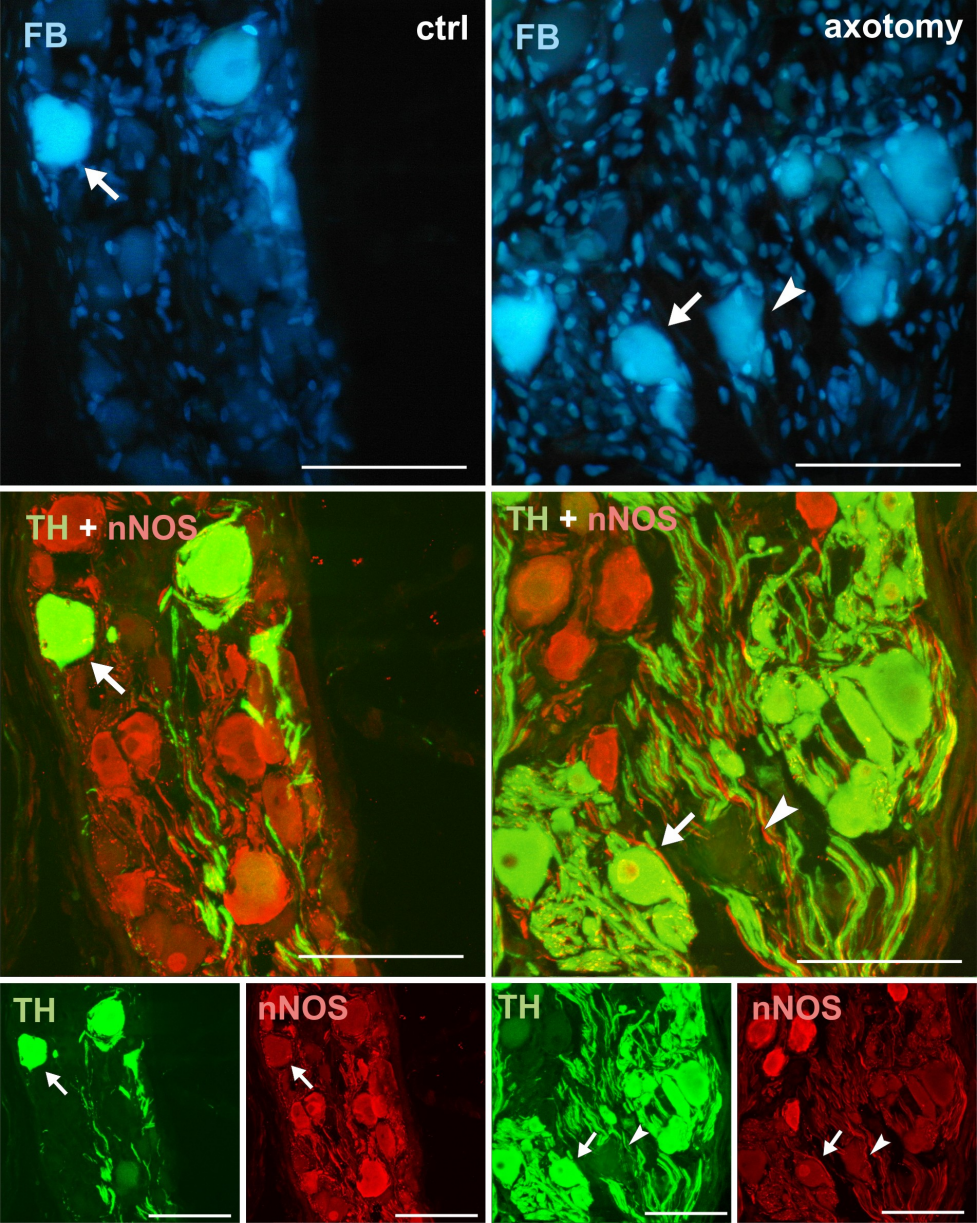
Changes in the expression of nNOS in PCG neurons after hysterectomy. In the control group all FB + neurons contained TH and did not contain nNOS-immunoreactivity (arrows). After hysterectomy some neurons not containing TH- or nNOS-immunoreactivity were observed (arrowhead). Scale bars 50 μm.

**Fig 10:**
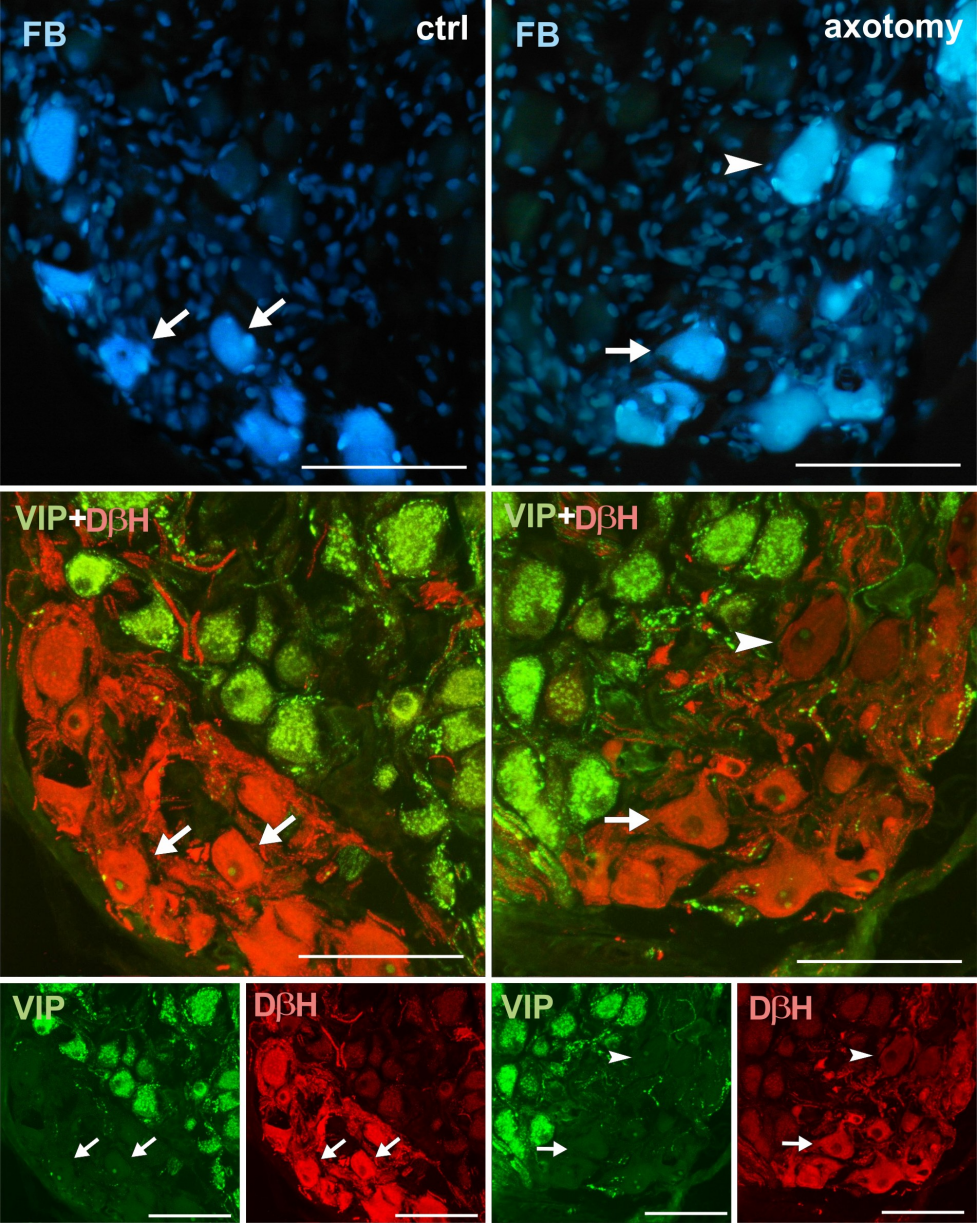
Changes in the expression of VIP in PCG neurons after hysterectomy. In the control group all FB+ neurons contained DβH and did not contain VIP-immunoreactivity (arrows). After hysterectomy FB+ neurons not containing DβH- or VIP-immunoreactivity were observed (arrowhead). Scale bars 50 μm.

**Fig 11:**
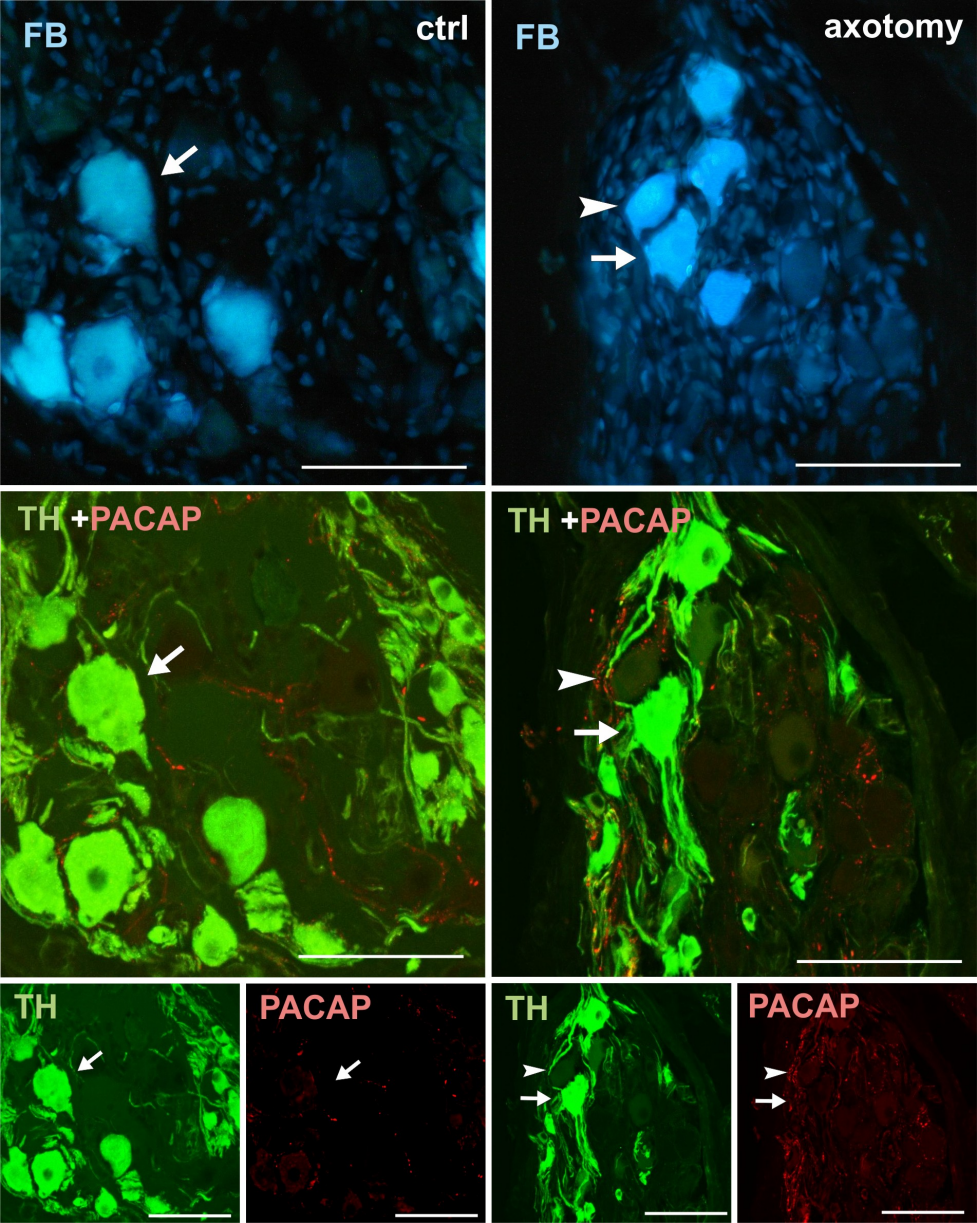
Changes in the expression of PACAP in PCG neurons after hysterectomy. In the control group all FB+ neurons contained TH- and did not contain PACAP-immunoreactivity (arrows). After hysterectomy FB+ neurons not containing TH- or VIP-immunoreactivity were observed (arrowhead). Scale bars 50 μm.

**Fig 12:**
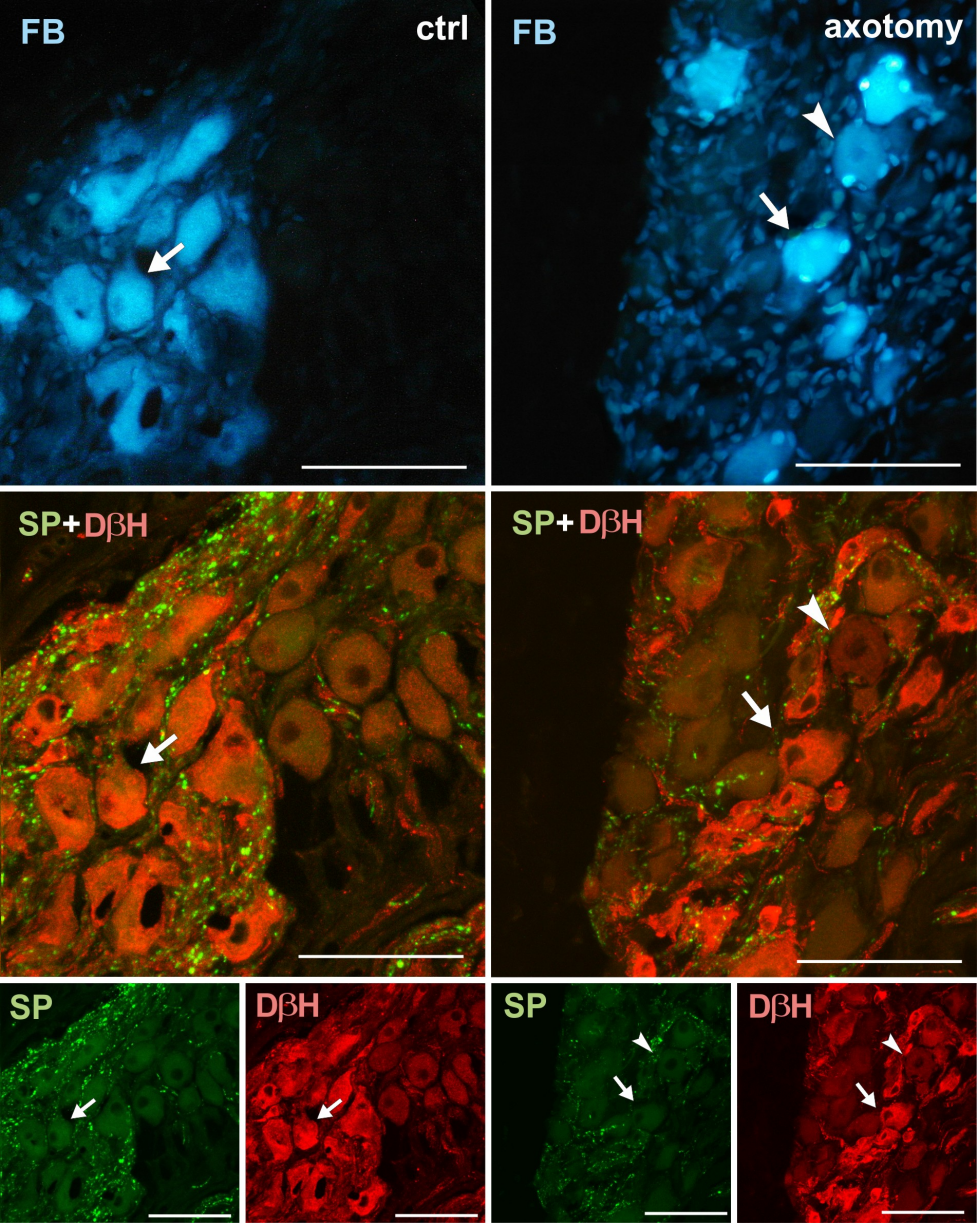
Changes in the expression of SP in PCG neurons after hysterectomy. In the control group all FB+ neurons contained DβH- and did not contain SP-immunoreactivity (arrows). After hysterectomy FB+ neurons not containing DβH- or SP-immunoreactivity were observed (arrowhead). Scale bars 50 μm.

Virtually all FB+ neurons both in the right and left PCG of the control animals showed immunoreactivity for tyrosine hydroxylase (TH) and dopamine beta hydroxylase (DβH) (Figs 2A and 2B, 3–12). In PCG of animals after hysterectomy, a significant decrease in the number of FB+/TH+ neurons was observed (61.498% ± 2.942% in right and 94.44% ± 1.375% in left PCG) and FB+/DβH + (65.48% ± 1.545% in right and 95.96% ± 0.854% in left PCG, Fig 2A and 2B). In contrast to the control group, the intensity of immunofluorescence in perikaryons varied, ranging from strong to barely visible.

Galanin immunoreactivity was absent in the FB+ neurons in the control group. Interestingly, after hysterectomy, galanin immunoreactivity was observed in 42.48% ± 2.397% of FB+ neurons in the right and 8.22% ± 0.421% of FB+ neurons in the left PCG (Figs 2C and 4).

NPY immunoreactivity was present in a smaller number of FB+ neurons (63.22% ± 1.934% in the right and 65.320% ± 1.075% in the left PCG of control animals). After hysterectomy, a significant increase in the number of FB+/NPY+ neurons was observed in the right (76.32% ± 1.46%) and insignificant (68.32% ± 2.259%) in the left PCG, Fig 2D). Almost all FB+/TH- neurons showed relatively strong NPY immunoreactivity (Fig 5).

In control group somatostatin-immunoreactivity was observed in 7.76% ± 0.731% of FB+ neurons in right and 7.84% ± 0.754% in left PCG. The number of somatostatin-immunoreactive neurons did not change significantly after hysterectomy and was respectively, 8.02% ± 0.689% and 8.08% ± 0.574% for right and left PCG (Figs 2E and 6).

No other immunoreactivities in FB+ neurons were found in both the control animals and in animals after hysterectomy. However, ChAT-, VAChT-, nNOS- and VIP- immunoreactive FB-negative perikarya in the PCGs were observed. PACAP and SP immunoreactivity were visible only in the nerve fibres of the PCGs (Figs 7–12).

## Discussion

### TH, DβH

A typical reaction of sympathetic neurons to axotomy is inhibition of the production of their main neurotransmitter, norepinephrine. This is related to the decrease in the expression of basic enzymes involved in its synthesis, namely TH and DβH [14, 26, 30–35]. This is probably due to the lack of access to NGF, because its administration to axotomized neurons prevents the decline in TH expression [14, 36]. The decrease of the expression of this enzyme in the neurons can also be caused by the administration of NGF-binding antibodies, which prevents uptake of NGF by nerve endings [37, 38].

In this study, as a result of cutting off a part of the axon of PCG neurons innervating the uterine horn, there was a decrease in the number of TH-immunoreactive neurons (in the right PCG from 100% to 61% and in left PCG from 100% to 94%) and DβH (in the right PCG from 100% to 65% and in left PCG from 100% to 96%). In other studies in pigs, after axotomy the number of TH+ neurons in inferior mesenteric ganglion (IMG) innervating the uterine horn decreased on the ipsilateral side from 96% to 41%, and on the contralateral side from 93% to 88% [26]. Axotomized neurons innervating the cervix showed a greater decrease in TH expression (their number decreased from 95% to 33%) [26]. Similar decrease in the expression of TH and DβH was observed in sympathetic chain ganglia (SChG) [39] and IMG [40] neurons supplying colon in the pig. Differences in the number of neurons responding to axotomy with decrease of TH expression can be explained by the different sensitivity of neurons to the lack of neurotrophins. As a result of the excision of the uterine horn, inflammation develops in the uterine corner stump. Inflammatory macrophages that enter the healing wound may stimulate tissue to increase NGF production [41]. Perhaps, for some neurons, the amount of NGF produced at the axon intersection is sufficient to stop the phenotype change. It is puzzling that such a weak reaction occurs in the neurons of the left PCG. It is possible that the neurons in the left PCG are mainly responsible for the innervation of the left corner of the uterus. However, some of them could have collateral axon which project to the right uterine horn, where the neuronsl tracer originates from. After axotomy collateral axon innervating the left horn may take the amount of trophic factors sufficient for the proper functioning of the neuron.

### ChAT, VAChT

Cholinergic nerve fibers were described in the uterus of many species, including domestic pig [42]. However, our study did not show any cholinergic neurons innervating the uterine horn in the PCG of the pig. It is possible that cholinergic neurons innervating uterine horn are present in other small clusters of neurons of the pelvic plexus, which are located in the pelvic cavity at a greater distance from the cervix and vagina. Also, after axotomy, neurons innervation uterine horn did not change their phenotype to cholinergic. This is consistent with the results of other studies, because so far there are no reports on the changes to the cholinergic phenotype in sympathetic neurons after axotomy [26, 39].

### nNOS

Studies in the rat indicate that the nitrergic nerve fibers supplying the uterine horn can derive from PCG [43]. However, the results of our study suggest that porcine PCG neurons do not participate in the innervation of the uterine horn. There are no data on changes in nNOS expression in pelvic neurons after axotomy.

A slight increase in the occurrence of nNOS in adrenergic neurons after axotomy, not exceeding 2% of all neurons in the ganglion, was described only in the superior cervical ganglion (SCG) of rat [44].

### Galanin

Several studies showed induction of galanin expression after axotomy. Already 48h after axonal injury expression of galanin increases 200-fold in rat SCG neurons [45]. The function of galanin in the reaction to axotomy is not well understood. It is known that galanin is transported in the damaged axon anterogradely to the lesion site [46–48], but there is no direct evidence that it plays any role in axonal regeneration. It is believed that galanin may affect regeneration by stimulating non-neuronal cells at the site of injury [49]. The mechanism of up-regulation of galanin expression after axotomy is not fully understood. However, it is known that trophic factors deprivation is responsible for the increase of the expression of galanin. This thesis is supported by studies with substances blocking axonal transport, eg colchicine, which led to the increase of galanin expression [50].

Our study indicates that galanin was absent in PCG neurons innervating the horn of the porcine uterus, which is consistent with other studies in this species [2]. However, after axotomy, galanin expression appears in about 42% of FB+ neurons on the right and 8% in the left PCG. Data obtained from studies on rat SCG neurons show that about 80% of neurons of this ganglion respond to axotomy by an increase in galanin expression [45, 51]. Also studies using pigs showed dramatic upregulation of galanin expression after axotomy in the neurons supplying the colon and pelvic cavity organs [29, 39, 52, 53].

### NPY

The expression of some other neuropeptides also changes in neurons in response to axonal injury. One of these neuropeptides is NPY, whose presence in PCG neurons innervating the uterus was found in the rat [54], guinea pig [55], and pig [2]. The character of the changes in the expression of this neuropeptide after axotomy is well known in the neurons of the DRG, where NPY is not expressed under physiological conditions. After axotomy NPY expression appears, especially in the large diameter neuronal population [56–58]. In sympathetic neurons of the anterior cervical ganglion more than half of the neurons expressed NPY under physiological conditions [59]. Rodent studies show that after axotomy the expression of NPY is reduced in the sympathetic neurons [60].

Our results showed that all PCG neurons of pig innervating the uterine horn are adrenergic. Therefore, it would be expected that these neurons will show similar response to axotomy as described above SCG neurons of the rat. However, our results showed the increase in the number of NPY+ neurons after axotomy (from 63% to 76% in the right and 65% to the 68% in the left ganglion). Additionally, vast majority of FB+/TH- neurons exhibit a strong immunofluorescence to NPY what suggests that the expression of this neuropeptide in porcine PCG increases after axotomy. The observed changes do not necessarily indicate an increase in expression of NPY gene. The accumulation of NPY in PCG nerve cell body detected by immunohistochemistry may be the result of selective blockage of axonal transport rather than increase in the production of this neuropeptide. This thesis is supported by the results of Kroesen’s studies [61], where in the SCG of the rat after nerve damage the amount of peptide was doubled, however the mRNA level for NPY was lower than in the control animals. Differences between the results presented in presented study and the results of other authors may also be the result of different experimental model. In the studies cited above, axons were cut at a short distance from the nerve cell body. In contrast, in our study the lesion was located at a substantial distance from the nerve cell body, which may be important for the response of the nerve cell to axotomy. In addition, adrenergic neurons of rodents can respond in a different way to axotomy than neurons of pig. Experiments using domestic pig revealed that adrenergic neurons of IMG respond in a way similar to neurons in the present study. In these studies, the number of NPY+ neurons increased [29, 53], but no increase in mRNA for NPY was detected [29].

### Somatostatin

In this study, approximately 8% of PCG neurons innervating the uterus horn contained somatostatin. There is no data available showing the presence of somatostatin in PCG neurons innervating the uterus. There are only reports on the presence of somatostatin in PCG neurons that innervate the ovary of the pig [53]. Our studies showed that after axotomy the number of somatostatin-immunoreactive neurons innervating the uterine horn did not change significantly. The decrease in the expression of somatostatin after axon damage was reported only in the sensory neurons [62–64]. In the case of sympathetic neurons, there have been reports of increased expression of somatostatin after axotomy in rat SCG neurons [60] and IMG [53] and SChG of the domestic pig [39].

### VIP, PACAP

Other neuropeptides for which expression changes induced by axotomy have been described are VIP and PACAP. PCG is a postulated source of VIP-immunoreactive fibers that innervate the uterus of many animal and humans [65].

There are many studies showing substantial increase in VIP and PACAP expression after axotomy in sympathetic neurons [44, 51, 66–71]. The role of the VIP and PACAP in neurons after damage to their axons is not fully understood. VIP and PACAP activate adenyl cyclase, which increases cAMP levels in the neuron [72]. The neuroprotective effects of these peptides are also well known. VIP increases neuroblast survival in rat sympathetic neuron culture and stimulates mitotic division and axonal growth [73].

However, in this study, those neuropeptides were not found in PCG neurons innervating uterine horn, also no VIP nor PACAP were found in these neurons after axotomy. Also other studies in pig did not show change in the VIP synthesis rate in neurons after axotomy [29, 39, 74]. It seems that reaction of these neuropeptides to axotomy may be species-specific process.

### SP

SP is a neuropeptide that is expressed mainly in sensory neurons. This agrees with the results of our study, where no SP-immunoreactive neurons were observed among the FB+ neurons located in PCG.

No changes in SP expression were found in our studies. Data on the increase in synthesis of this neuropeptide after axotomy refer only to the SCG [60, 68, 70]. There are no data about changes in the expression of SP in neurons of the pelvic ganglia. Studies on axotomized IMG neurons innervating the uterus of the pig showed that SP expression did not change also [29].

## Conclusion

Our study showed that virtually all neurons of the porcine PCG innervating the uterine horn are adrenergic and we did not confirm that PCG is the source of cholinergic fibers innervating uterine horn of the domestic pig. After axotomy there was a decrease in the expression of catecholamine pathway enzymes (TH, DβH) and a strong increase in galanin expression. The increase in the number of NPY-IR neurons in the ganglia after axotomy was observed. There was no change in the expression of other studied substances in the PCG neurons innervation uterine horn, which was often found in rodents studies. This indicates that neurons can respond to damage in a species-specific way and studies of nervous system damage should not be conducted with only single model organism. This heterogeneity of response to injury may point to variable ability of different species to undergo regeneration or respond to pro-regenerative therapies.
