# Correction: Effect of partial hysterectomy on the neurons of the paracervical ganglion (PCG) of the pig

**DOI:** 10.1371/journal.pone.0253676

**Published:** 2021-06-17

**Authors:** Piotr Podlasz, Krzysztof Wasowicz

The following information is missing from the Funding statement: This project was financially supported by the Minister of Science and Higher Education in the range of the program entitled "Regional Initiative of Excellence" for the years 2019–2022, Project No. 010/RID/2018/19, amount of funding 12.000.000 PLN.
